# Bacteriophage lysins: a promising strategy for combating foodborne pathogens in food safety

**DOI:** 10.3389/fmicb.2026.1892513

**Published:** 2026-07-17

**Authors:** Jiaxin Wei, Yuying Zhang, Yubao Li, Lei Wang

**Affiliations:** 1 School of Pharmaceutical Sciences and Food Engineering, Liaocheng University, Liaocheng, China; 2 Shandong Key Laboratory of Applied Technology for Protein and Peptide Drugs, Liaocheng University, Liaocheng, China

**Keywords:** bacteriophage, biological control, endolysin, food safety, foodborne pathogens, phage lytic enzyme

## Abstract

Food safety is a major public concern, with foodborne pathogens such as *Staphylococcus aureus*, *Listeria monocytogenes*, *Clostridium perfringens*, and *Vibrio parahaemolyticus* causing millions of infections and significant economic losses annually. Phage and endolysin therapies have emerged as promising environmentally friendly strategies for pathogen control. Bacteriophage lytic enzymes, which are proteins produced during the late stage of phage infection, offer advantages over whole phages, including rapid bactericidal action, a broad antibacterial spectrum, and a low risk of inducing resistance. This review summarizes the structure, classification, and lytic mechanism of phage lysins, as well as their application in combating foodborne pathogens across various food matrices, including meat, dairy products, agricultural produce, and seafood. Furthermore, these lysins have demonstrated promising potential in biofilm removal. Meanwhile, this review discusses multiple improvement strategies that have been developed to enhance the activity of lysins against both Gram-positive and Gram-negative bacteria. These include domain truncation, site-directed mutagenesis, and domain shuffling to overcome insufficient stability and suboptimal activity in food environments, as well as co-administration with outer membrane permeabilizers, encapsulation and engineered lysins (e.g., Artilysins, Innolysins, and Lysocins) to breach the outer membrane barrier of Gram-negative bacteria. Furthermore, the introduction of computer-aided and bioinformatics tools has provided new avenues for the high-throughput screening and rational design of lysins. Although challenges such as low screening efficiency, unstable activity in food matrices, non-uniform efficacy endpoints, regulatory data requirements, high production costs, and limited consumer acceptance persist, lysins, as green and efficient natural antimicrobial proteins, still exhibit broad application prospects in food biocontrol.

## 1 Introduction

Food safety is a critical concern in modern society. Pathogen contamination and food deterioration pose significant challenges to global public health and food security. Foodborne pathogens, which primarily consist of bacteria, viruses, parasites and fungi, are widespread in the natural environment. Currently, the most prevalent foodborne pathogens are bacteria, such as *Salmonella* spp.*, Escherichia coli, Clostridium botulinum, Staphylococcus aureus, Listeria monocytogenes, Campylobacter* spp., and *Vibrio parahaemolyticus* (Bintsis, 2017; Davydova et al., 2025). These pathogenic bacteria are transmitted through multiple routes, including contaminated food or water, contact, and person-to-person spread. Foodborne diseases caused by pathogenic bacteria typically present with symptoms such as fever, vomiting, abdominal pain, and diarrhea. There are two primary pathways by which foodborne infections develop. The first is intoxication, in which a pathogen on the food surface or inside the food product produces a toxin, which then enters the organism with the meal and affects its metabolism. The second is direct infection, in which a pathogen ingested with food can adapt and multiply within cells (Hyla et al., 2022).

**Figure 1:**
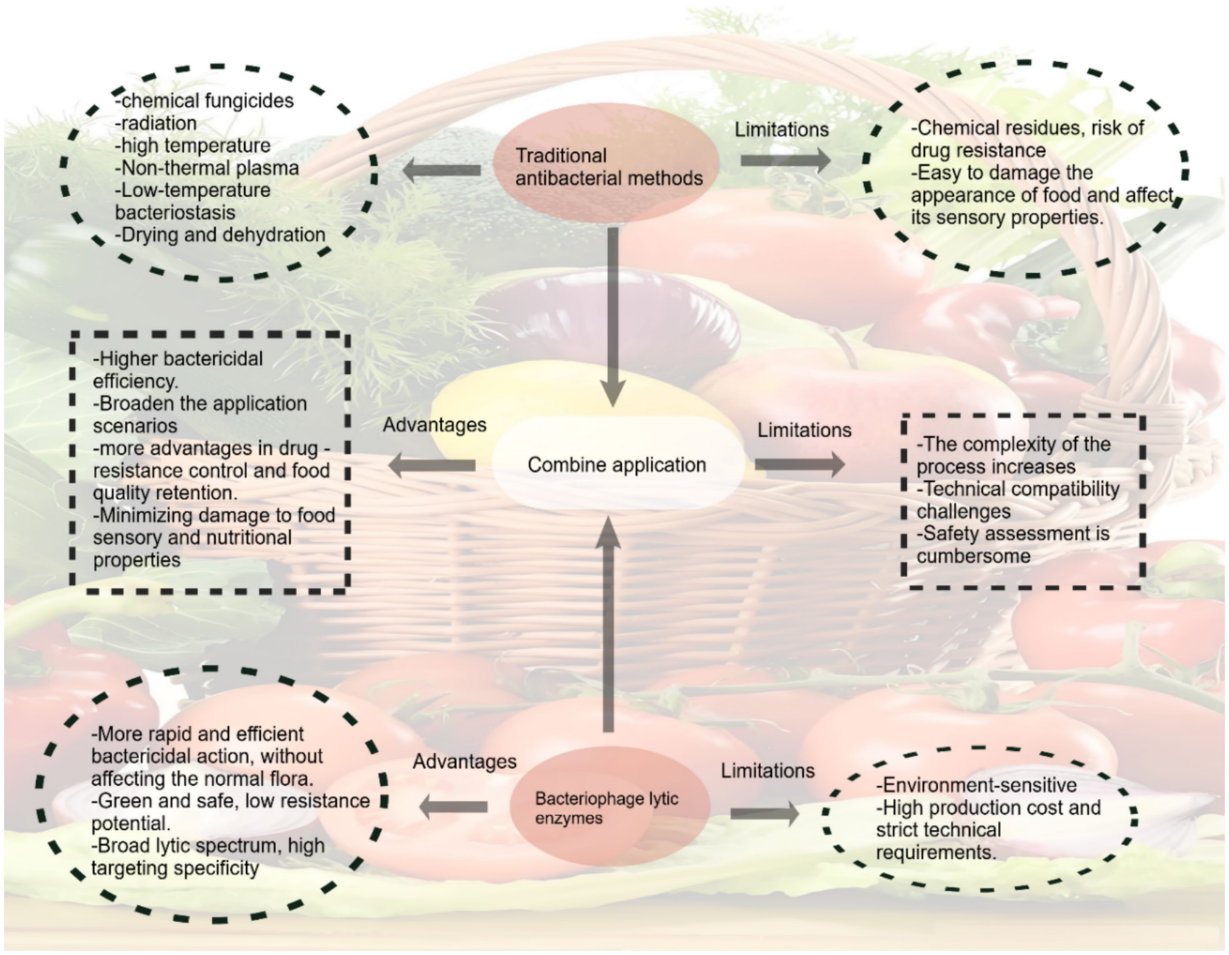
Synergistic food safety: comparison and combination of conventional antibacterial methods and phage lytic enzymes.

In the traditional food industry, the most common methods of prevention and control involve using chemical disinfectants such as chlorine-based compounds, organic acids and quaternary ammonium salts. Although they are effective at inhibiting pathogenic bacteria in the short term, prolonged use can promote bacterial resistance and pose significant risks to ecological and human health. As illustrated in Figure 1, traditional antimicrobial methods and phage lytic enzymes differ fundamentally in their mechanisms and safety profiles. According to a report by the World Health Organization (2022) on the global burden of foodborne diseases, contaminated food causes approximately 600 million illnesses annually, affecting almost one in ten people worldwide, and results in 420,000 deaths annually. In recent years, there has been an increasing trend towards using methods such as irradiation, high-pressure processing, and non-thermal plasma (NTP) to inactivate foodborne pathogens. However, these methods are highly likely to affect the appearance and sensory properties of food (Xu, 2021). Recently, many natural compounds have exhibited antimicrobial activity. For example, gallic acid disrupts MRSA biofilms by inhibiting bacterial adhesion and PIA synthesis, thereby advancing the development of green antimicrobial strategies (Sang et al., 2024). Currently, there is considerable interest in the potential of bacteriophages and their endolysins for combating drug-resistant bacteria. The bacteriophage has received GRAS (Generally Recognised as Safe) status from the US FDA and has been approved as a food preservative in the US, Canada, the EU, Australia and New Zealand, among other regions (O’Sullivan et al., 2019). Compared to bacteriophages, endolysins are considered safer for use in food because they have a unique specific mode of action that neither triggers gene transfer nor leads to the emergence of resistant bacteria (Ho et al., 2022; Li et al., 2025b). Endolysins have been shown to be effective against *V. parahaemolyticus, S. aureus, and C. perfringens* in food industry applications (see Tables 1, 2).

**Table 1:** Applications of natural endolysins in food matrices: treatment conditions and reported efficacy endpoints.

Pathogen	Endolysins	Application	Conditions and endpoint	References
*Staphylococcus aureus*	LysP152	Pork	At 140 μg/cm^3^ and 28 °C for 3 h, LysP152 reduced bacterial counts in meat by 2.06 log₁₀ CFU/mL.	Li et al. (2025a)
	LytN	Pork and food contact surfaces	On both pork and stainless steel surfaces, endolysin LytN (50 μg/mL, 37 °C, 6 h) exhibited greater bactericidal efficacy against MRSA than its parent phage, achieving reductions of 3.5 log and 4.3 log, respectively.	Zhang F. et al. (2026)
*Salmonella* spp.	LysPd078	Mung beans	At concentrations as low as 50 μg/mL, this treatment achieved a 4- to 5-log reduction in bacterial counts on contaminated mung bean seeds.	Yao et al. (2025)
	LysP53	Lettuce	LysP53 at 4 μM displays potent bactericidal activity against *Salmonella*, reducing bacterial counts on fresh lettuce by 90% within 30 min.	Li et al. (2023)
*Listeria monocytogenes*	LysP70	Lettuce and milk	Following LysP70 treatment at a concentration of 400 μg/mL, the maximum reductions in *L. monocytogenes* counts were 1.9 log₁₀ CFU/mL in milk and 1.33 log₁₀ CFU/cm^2^ on lettuce, respectively.	Zhang et al. (2025)
	Lys-LP-XY2101	Apple juice, skim milk, pork ham, and spiced beef	*L. monocytogenes* was reduced to undetectable levels in various food matrices following treatment at 4°C, 16 °C, or 25 °C.	Ji Q. et al. (2026)
	PlyP40/PlyPSA	Queso fresco	At 250 μg/g in Queso Fresco (4 °C, 28 d), PlyP40 reduced *L. monocytogenes* by approximately 0.7 log, outperforming PlyPSA, which was inactive after day 14.	Holle and Miller (2021)
*Clostridium perfringens*	PlyDolk21	Beef soup and milk	In beef broth, treatment with 0.1–0.5 μM PlyDolk21 at 25 °C for 24 h reduced viable *C. perfringens* counts by 3–4 log, whereas in milk, a concentration of 1.5 μM was required to achieve a 3-log reduction under the same conditions.	Seo et al. (2025)
	Lys19	Lettuce and raw pork	Treatment with Lys19 (100 μg/mL) resulted in an 88.75% reduction of *Clostridium perfringens* on lettuce within 15 min, and an approximately 84.65% reduction in raw pork after 12 h at 4 °C.	Ji Y. et al. (2026)
*Escherichia coli*	PlyEc2	Lettuce	It reduces Shiga toxin-producing *E. coli* O157:H7 contamination on lettuce by 99.7%.	Xu S. et al. (2021)
*Bacillus cereus*	LysDLn1	Milk	Treatment with 46 μg/mL endolysin resulted in a 0.8 log_10_ CFU/mL reduction in viable bacteria after 3 h, with the effect persisting to a 4.7 log_10_ CFU/mL reduction at 24 h.	Li et al. (2022)
*Vibrio parahaemolyticus*	Lys59	Biofilm	At 50 μg/mL, endolysin Lys59 penetrates the outer membrane of *V. parahaemolyticus* without the aid of a permeabilizer, achieving a 68.2% reduction in OD₆₀₀.	Liu et al. (2025)
*Streptococcus suis*	Lys572	Pork	During storage at 4°C for 48 h, 50 μg/mL of Lys572 effectively reduces *S. suis* in pork by approximately 2 log_10_ CFU/mL. Lys572 also effectively reduces bacterial loads during thawing at 4 °C and 30 °C, achieving a maximum reduction of 2.0–2.2 log_10_ CFU/mL.	Ning et al. (2026)
*P. fluorescens*	LysPFX32	Pork	Treatment with 95 μg/mL LysPFX32 reduced *P. fluorescens* counts in pork by approximately 2.47 log (30 °C, 24 h) and extended the refrigerated shelf life (4 °C) by about 2 days.	Guan et al. (2025)

Outcomes are presented as log10 CFU reductions whenever reported in the source study. Percentage reductions, complete inactivation, or qualitative matrix effects are retained when log10 CFU data were not available. Direct comparison across studies should consider differences in inoculum, dose, temperature, exposure time, food matrix, and storage conditions.

**Table 2:** Synergistic application effects of endolysins: treatment conditions and reported endpoints.

Endolysin	Synergy with	Target pathogen	Conditions and endpoint	References
LysSA97	Carvacrol	*Staphylococcus aureus*	In skimmed milk, the combination of 1.88 μM LysSA97 and 6.66 mM carvacrol reduced viable *S. aureus* counts to below the detection limit within 3 h. In lean beef, the same combination (with 18.8 μM LysSA97) achieved a reduction of 2.1 ± 0.5 log CFU/cm^2^ over the same period.	Chang et al. (2017)
PlyP825	HPP	*Listeria monocytogenes*	Combined endolysin (PlyP825) treatment at 400 MPa reduced *L. monocytogenes* counts by approximately 3.9 log in milk (3.4 μg/mL PlyP825) and by approximately 4.1 log in mozzarella cheese (34 μg/mL PlyP825).	Misiou et al. (2018)
LysPB32	Essential oils	*Salmonella* spp.	Combined treatment with LysPB32 and essential oil reduced bacterial counts in cooked ground beef by more than 2 log units after 24 h of storage at 37 °C and after 7 days of refrigeration.	Kim et al. (2024)
rLysJNwz	EDTA	*Salmonella* spp.	The synergistic effect with EDTA exhibits bactericidal activity against multiple Gram-negative bacteria, achieving reductions of over 86.7% on eggs and 86.5% on lettuce following a 60-min treatment.	Shen et al. (2023)
LysPEF1-1	Cinnamon bark essential oil	*Listeria monocytogenes*, *Enterococcus faecalis*	Treatment with 50 μg/mL LysPEF1-1 combined with 0.05% C.b.EO reduced bacterial counts on cheese surfaces by 0.6 log at 25 °C and by 1.2 log at 37 °C. Furthermore, this combination demonstrated high efficacy in eliminating mixed-species biofilms on various foods and food-contact surfaces.	Wang et al. (2025)
LysH5	Nisin bacteriocin	*Staphylococcus aureus*	In pasteurized milk, the combination of LysH5 (7.5–15 U/mL) and nisin (0.37–0.75 μg/mL) completely eradicated *S. aureus* within 6 h, achieving a 4- to 6-log reduction in viable counts compared with the untreated control.	García et al. (2010)
SAP8	Nisin	*Staphylococcus aureus*	Combination treatment with 0.01 μM SAP8 endolysin and 18 IU/mL nisin reduced viable *S. aureus* counts from 5.90 to 3.85 log CFU/mL within 3 h, and halved the bacterial population on lettuce within 6 h, indicating a strong synergistic effect.	Kim S. G. et al. (2022)
LysRODIΔAmi	Nisin	*Staphylococcus aureus*	In laboratory-scale cheese production, endolysin combined with nisin was more effective in reducing *S. aureus* counts than endolysin alone.	Youssef et al. (2023)
cpp-lys	EDTA	*Clostridium perfringens*	The combination of 10 μg cpp-lys and 5 mM EDTA reduced bacterial counts on lettuce by approximately 4.6 log.	Zhao et al. (2023)
lysAB-vT2-fusion	Colistin	*Acinetobacter baumannii*	The combination of the lysAB-vT2 fusion protein and colistin exhibits synergistic antibacterial activity against *A. baumannii*, achieving up to 95.8% inhibition against susceptible strains.	Sitthisak et al. (2023)
Lyz_V_pgp60	Gentamicin	*Vibrio parahaemolyticus*	Combined treatment with Lyz_V_pgp60 (100 μg/mL) and gentamicin increased the bactericidal rate against *V. parahaemolyticus* from 13 to 43.42% within 30 min.	Liu J. et al. (2024)
LysEP114, LysEP135	Lactic acid	*Escherichia coli*	With 50 mM lactic acid, LysEP114 and LysEP135 reduced biofilm cell counts from 7.3 to 4.1 and 2.2 log CFU/mL, respectively.	Hasan et al. (2024)

Synergistic outcomes depend on the lysin dose, co-treatment concentration, food matrix, exposure time, and temperature. Biofilm biomass reduction and viable-cell reduction are distinct endpoints and should be interpreted separately.

The combination of restrictions on antibiotic use and rising consumer demand for safe, sustainable food has created an urgent need for efficient and environmentally friendly biocontrol technologies. In recent years, endolysins have attracted increasing attention. As lytic proteins produced by bacteriophages, they degrade peptidoglycan in bacterial cell walls, thereby inducing lysis. Due to their potent lytic activity and high host specificity, these enzymes are both environmentally benign and safe, exhibiting a minimal propensity to induce resistance (Sharma et al., 2018; Xu, 2021). Thus, they have great potential for preventing and controlling the contamination of food by pathogenic bacteria, as well as the development of antimicrobial resistance. This review summarizes recent advances in using phage lysins to control foodborne pathogens, describes methods to improve their effectiveness against Gram-positive and Gram-negative bacteria, and examines their potential uses in the food industry.

## 2 Bacteriophages and phage lysins

Bacteriophages were discovered over a century ago. In 1915, the British pathologist Frederick Twort observed a “glassy transformation” in *Micrococcus* colonies. Two years later, the French-Canadian microbiologist Félix d’Hérelle isolated an antimicrobial agent active against *Shigella* and named it “bacteriophage,” meaning “bacteria eater” (Salmond and Fineran, 2015). It is estimated that there are around 10^31^ bacteriophages in the global population (Greber, 2019). Bacteriophages have a relatively simple structure consisting of nucleic acid surrounded by a protein capsid. Some more complex bacteriophages also contain a lipid envelope. Morphologically, bacteriophages fall into three categories: tailed, tailless and filamentous. Bacteriophages are viruses that specifically infect bacteria and are the most abundant biological entities on Earth. They are found in soil, water, air, plants, and animals (Hatfull et al., 2022).

Bacteriophages primarily kill bacteria via two pathways: the lytic cycle and the lysogenic cycle. During the lytic cycle, virulent phages bind to the bacterial surface, inject their DNA, and exploit the host’s replication machinery to produce progeny, ultimately leading to cell lysis and release of new virions. By contrast, during the lysogenic cycle, temperate phages inject their DNA, which can either integrate into the host genome and replicate passively or switch to the lytic cycle under specific conditions (Kropinski, 2018; Nakayinga et al., 2021).

In 1958, Jacob and colleagues first reported that bacteriophages encode proteins capable of lysing bacteria, establishing the essential role of these proteins in the phage life cycle (Jacob and Fuerst, 1958). Subsequently, Freimer et al. (1959) demonstrated that these lytic proteins retain potent bactericidal activity even in their purified form. Since these seminal discoveries, numerous phage-derived lytic enzymes have been identified and explored for diverse biotechnological applications (Freimer et al., 1959). Lytic enzymes, also known as endolysins or enzymatic antibacterial agents, are specific hydrolases produced at the end of the phage lytic cycle. They promote the release of daughter phages by breaking down essential chemical bonds within the bacterial cell wall’s peptidoglycan, which ultimately results in the death of the bacterium. As novel antimicrobial agents, bacteriophage lytic enzymes offer several distinct advantages over bacteriophages:

① Bacteriophage lytic enzymes have a broader spectrum of activity and provide faster, more efficient bactericidal effects. Phages require adsorption, replication, and progeny release to function, whereas lytic enzymes directly degrade peptidoglycan in the bacterial cell wall, achieving rapid killing without replication. For example, LysSGF3 and LysP6 have a broader spectrum of activity than their parent phages (Wang et al., 2024; Chang et al., 2025).
② Combine precise targeting capability with high biological safety. Its targeting specificity derives from its ability to recognize the peptidoglycan domain within pathogen cell walls. As proteins, they can be degraded into small peptides and amino acids under both environmental and physiological conditions, leaving no chemical residues and exhibiting zero accumulation, thereby ensuring exceptional safety.
③ Endolysins are not only safe but also exhibit a low propensity to induce resistance (Nazir et al., 2023).
④ Possess a simple modular architecture comprising a catalytic domain and a binding domain. This structural organization facilitates its engineering, enabling the rapid generation of derivatives with broad-spectrum targeting, high activity, and enhanced stability through strategies such as domain replacement, site-directed mutagenesis, and combination with other antimicrobial agents.

## 3 Structural characteristics and classification of bacteriophage lysins

The structural characteristics of phage lysins are closely associated with the bacterial genera they target. Due to the fundamental differences in cell wall structure between Gram-positive and Gram-negative bacteria, the structural features of lysins that act on these two types of bacteria differ significantly (Gerstmans et al., 2018; Cui et al., 2025).

### 3.1 Bacterial cell wall structure and types of lytic enzyme action

**Figure 2:**
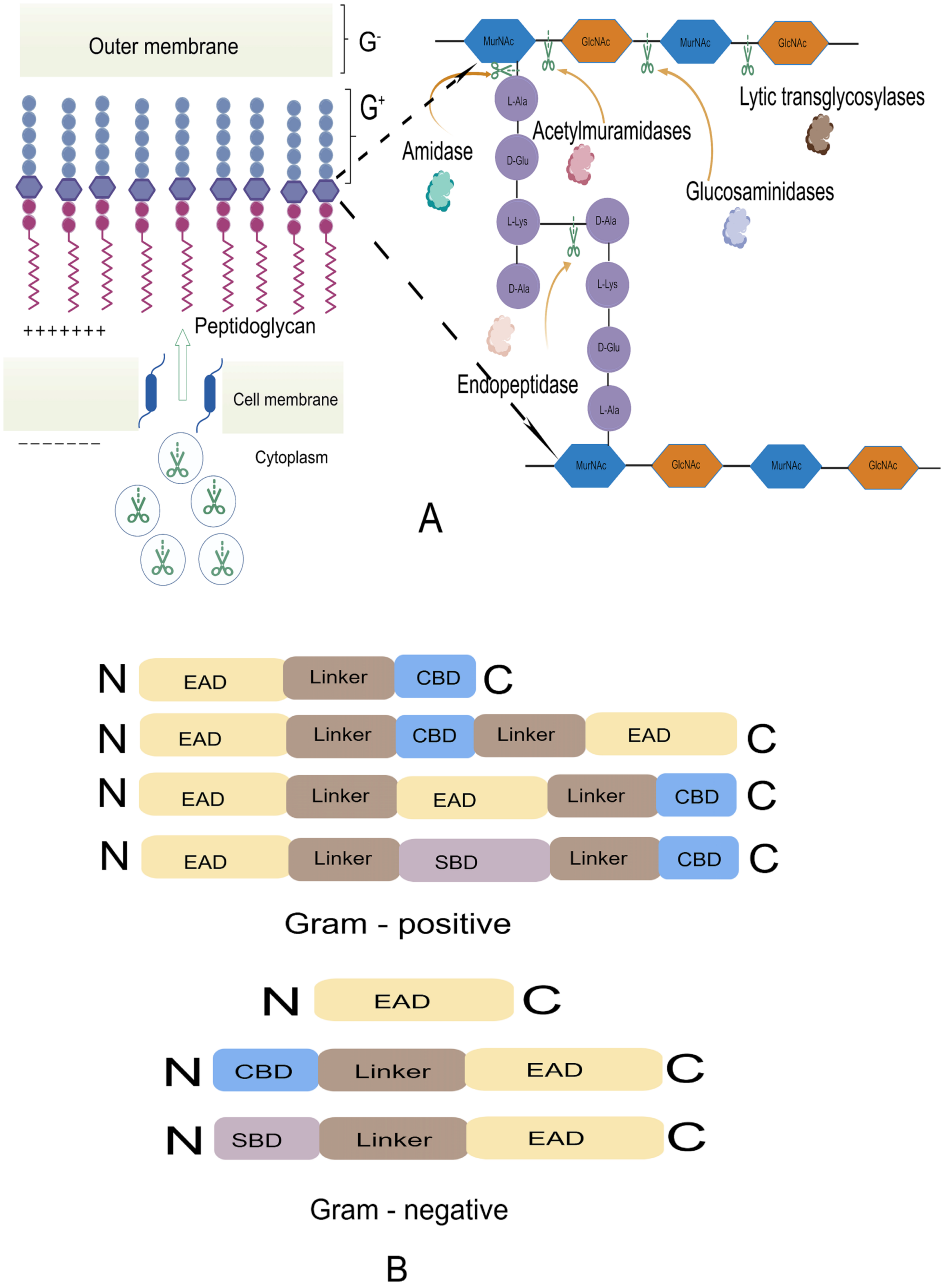
**(A)** Schematic representation of the peptidoglycan structure of gram-positive bacteria (L-lys type) and the cleavage sites of phage endolysins. **(B)** Schematic representation of the modular domain architectures of phage endolysins targeting gram-positive and gram-negative bacteria. EAD, Enzymatically Active Domain; CBD, Cell Wall Binding Domain.

The main component of the bacterial cell wall is peptidoglycan, a structurally complex polysaccharide-peptide complex that serves as the core structural material for maintaining cell shape and protecting against adverse environmental factors (Pazos and Peters, 2019). Its molecular structure comprises polysaccharide chains of alternating N-acetylglucosamine (NAG) and N-acetylmuramic acid (NAM) units, each NAM bearing a tetrapeptide side chain composed of L-alanine, D-glutamic acid, diaminopimelic acid (in Gram-negative bacteria) or L-lysine (in some Gram-positive bacteria), and D-alanine. These tetrapeptide side chains cross-link with those on adjacent polysaccharide chains via D-alanine, forming a network structure (Maneekul et al., 2024). Based on their peptidoglycan cleavage site and bond specificity, bacteriophage lytic enzymes fall into five main classes (Ajuebor et al., 2016; Gerstmans et al., 2018; Mondal et al., 2020; Abdelrahman et al., 2021):

① Endopeptidase: it cleaves the peptide cross-bridge by targeting the bond between L-lysine and D-alanine specifically.
② N-acetyl-*β*-D-muramidase (Lysozyme): it hydrolyses the glycosidic bond between N-acetylmuramic acid and N-acetylglucosamine, creating reducing NAM ends.
③ Lytic transglycosylase: it cleaves the same glycosidic bond as muramidase but does so via an intramolecular transglycosylation mechanism to form a 1,6-anhydro product.
④ N-acetyl-β-D-glucosaminidase: it hydrolyses the glycosidic bond between NAG and NAM at the non-reducing end.
⑤ N-acetylmuramoyl-L-alanine amidase: it hydrolyses the amide bond that links the N-acetylglucosamine sugar to the stem peptide (Figure 2A).

### 3.2 Structure of lysins targeting gram-positive bacteria

Most lytic enzymes derived from Gram-positive bacteriophages have a modular structure, with a molecular weight ranging from 25 to 40 kDa and exhibit amide-lyase activity. Generally speaking, the structure of these modular endolysins consists of two core domains: the N-terminal enzymatically active domain (EAD) and the C-terminal cell wall binding domain (CBD), which are linked by a short peptide chain (Schmelcher et al., 2012; Guo et al., 2021). The N-terminal domain serves as the catalytic domain of the enzyme, while the C-terminal cell wall-binding domain recognizes specific ligands within the bacterial cell wall (Kocot et al., 2023). Some lysins have been found to possess multiple CBDs or EADs, arranged in different orders (Yang et al., 2014). Furthermore, some lysins also contain an additional spore-binding domain, such as LysPBC2 from *Bacillus cereus* (Kong et al., 2019) (Figure 2B). Each domain affects antibacterial activity, and its specificity, activity, or solubility can be modulated through domain swapping (Gerstmans et al., 2018).

### 3.3 Structure of lysins targeting gram-negative bacteria

Gram-negative bacteriophage lysins typically exhibit a simple globular structure with a low molecular weight (15–20 kDa). Unlike the modular structure found in Gram-positive bacteria, they generally contain only a single EAD responsible for peptidoglycan hydrolysis, while most lack CBD modules. Additionally, their C- and N-termini are oriented oppositely to those of Gram-positive bacteria (Gerstmans et al., 2018; Liu et al., 2023; Kocot et al., 2023). However, a few lysins also adopt a modular structure. For example, OBPgp279 from the *Pseudomonas fluorescens* phage OBP and AP3gp15 from the Burkholderia phage AP3 exhibit a modular architecture consisting of an N-terminal CBD and a C-terminal EAD (Maciejewska et al., 2017; Walmagh et al., 2012). Recent research has shown that LysCPD7, an endolysin from *C. perfringens* phage CPD7, lacks a CBD domain but contains a spore-binding domain (SBD) (Ha et al., 2026) (Figure 2B).

## 4 Mechanism of action of lytic enzymes

**Figure 3:**
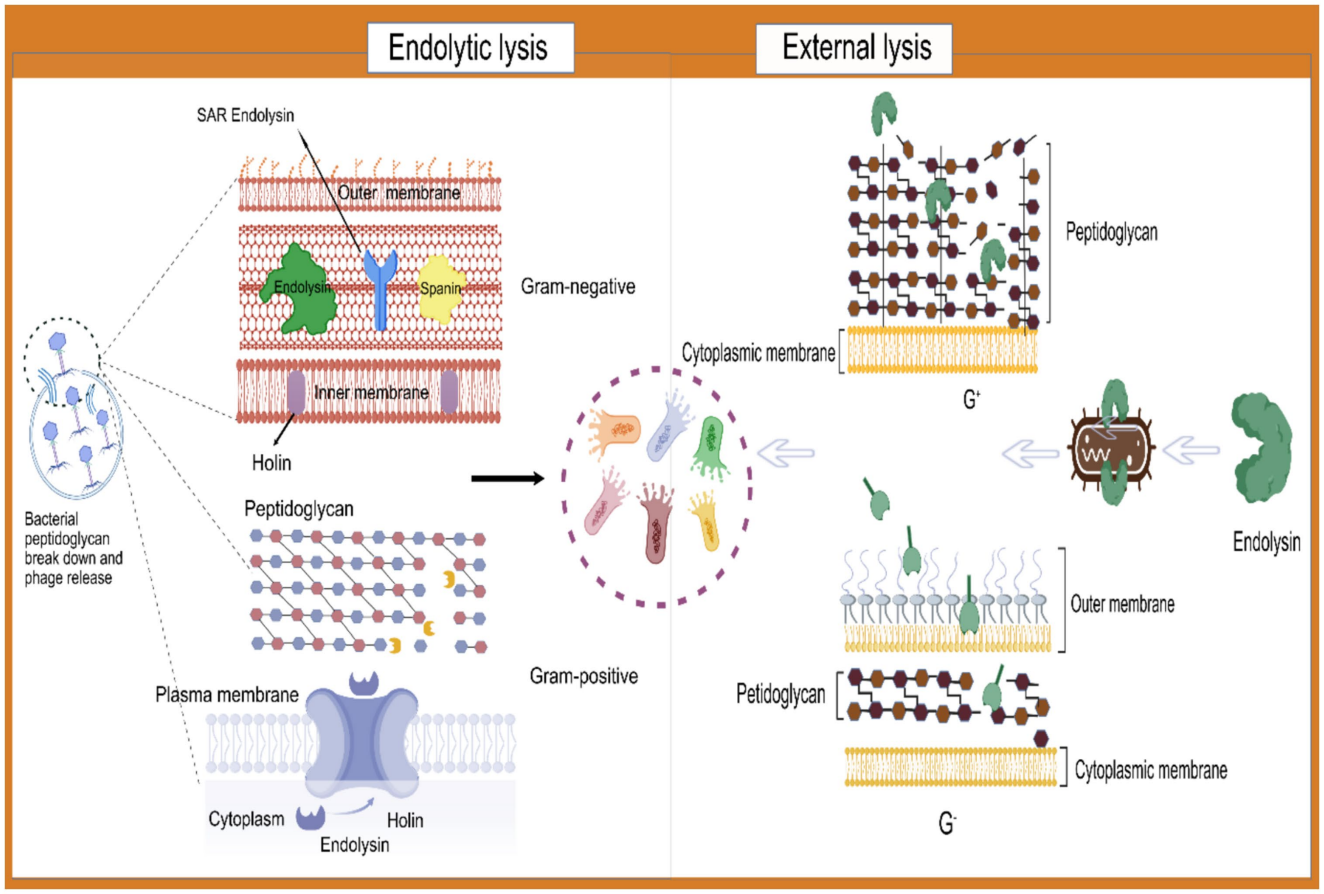
Schematic representation of endolysin-mediated bacterial lysis. (Left) Endolysis: holin oligomerizes to form pores in the inner membrane, allowing endolysin access to the peptidoglycan layer. Spanin complexes subsequently disrupt the outer membrane, facilitating cell lysis in Gram-negative bacteria. (Right) Exolysis: exogenous endolysins directly degrade the cell wall from the outside. This mechanism is effective against Gram-positive bacteria but requires outer membrane permeabilizers to act on gram-negative bacteria (Mondal et al., 2020; Nazir et al., 2023).

The action of phage lytic enzymes can be classified into two types: endolysis and exolysis (Abdelrahman et al., 2021; Cui et al., 2025). As illustrated in Figure 3, endolysis involves the coordinated action of endolysin, holin, and spanin to release progeny phages from within the bacterium. In contrast, exolysis occurs when exogenous endolysins directly degrade the cell wall from the outside. The core function of both is to employ endolysins for the specific degradation of bacterial peptidoglycan, thereby compromising cell wall integrity and ultimately causing the bacteria to lyse due to osmotic pressure. The differences are primarily evident in the triggering conditions, contributing factors and physiological functions.

Endolysis is the process by which a bacteriophage completes its life cycle within a host bacterium. Through the combined action of lysins and other associated proteins, lysis is initiated from within, serving as the primary mechanism for releasing progeny virions. This process involves three key proteins: endolysin, holin and spanin, which act on the bacterial peptidoglycan, outer membrane and inner membrane (Khan et al., 2023). Its mode of action depends on the host bacterium’s cell wall. In Gram-positive hosts, the holin-endolysin pathway is mainly relied upon. Holin forms channels on the intracellular membrane, allowing endolysin to cross the membrane and degrade peptidoglycan, thereby leading to cell lysis (Abdelrahman et al., 2021). In Gram-negative bacteria, two main lysis pathways have been characterized. The holin-endolysin-spanin pathway proceeds via holin-mediated inner membrane permeabilization, allowing endolysin access to the peptidoglycan, followed by outer membrane disruption through spanin complexes. In contrast, the SAR-type endolysin pathway involves an endolysin initially anchored to the inner membrane, which is released upon holin activation to degrade the peptidoglycan (Abdelrahman et al., 2021; Khan et al., 2023; Prajapati et al., 2025).

Exolysis means that lytic enzymes act directly on the surface of bacteria to initiate cell wall degradation from outside the bacteria. This process requires only the participation of exogenous lytic enzymes. Since Gram-positive bacteria lack an outer membrane barrier, exogenous endolysin can directly access and degrade the cell wall peptidoglycan, resulting in efficient bacterial lysis. However, due to the outer membrane barrier of Gram-negative bacteria, natural endolysins cannot directly induce lysis from without and typically require the assistance of outer membrane permeabilizing agents to exert their lytic effect (Lai et al., 2020).

## 5 Application of lytic enzymes against foodborne pathogens in food

**Figure 4:**
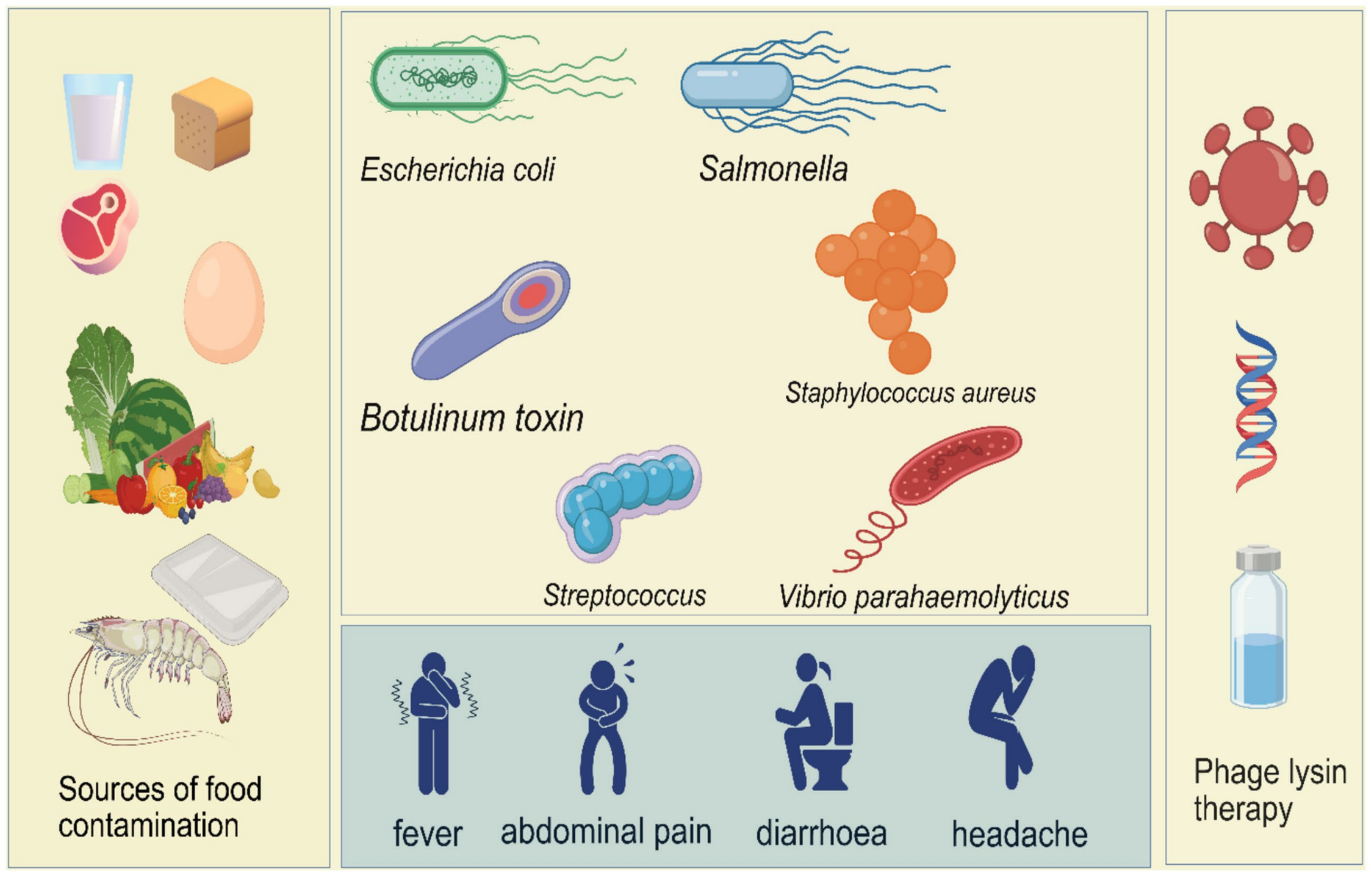
Schematic illustration of phage lysin applications for food safety control. Pathogen contamination occurs at multiple stages from farm to fork, leading to foodborne illnesses with clinical symptoms such as fever, vomiting, and diarrhea. Phage lysins can be applied as biocontrol agents during food processing, packaging, and storage to mitigate these risks.

The threat posed by foodborne pathogens is pervasive. Food is susceptible to pathogen growth and colonization throughout the entire farm-to-fork continuum, including transportation, storage, and processing. These invisible health threats compromise consumer well-being and can be life-threatening. As illustrated in Figure 4, phage lysins can be applied at multiple stages along the food supply chain to effectively control pathogens in meat, dairy, vegetable, and egg products, thereby preventing foodborne illnesses and their associated clinical symptoms. Foods of both animal and plant origin are rich in nutrients, such as proteins and moisture, which provide a favorable environment for pathogen growth and reproduction. Numerous studies have confirmed that endolysins show promising applications in both food types and represent highly promising biocontrol agents (see Table 1).

To improve comparability among application studies, antimicrobial outcomes in this review are reported as log10 CFU reductions whenever the original data permit. When studies reported percentage reduction, complete inactivation, sensory outcomes, or biofilm biomass rather than viable counts, these endpoints are retained as originally described and distinguished from culture-based log reductions. For food-matrix applications, the most informative experimental descriptors are the lysin dose, initial inoculum, treatment time, treatment temperature, food matrix, application mode, and evidence of pathogen regrowth during storage (Bai et al., 2019; Shannon et al., 2020; Guan et al., 2025).

### 5.1 Lytic enzymes against pathogenic bacteria in animal-derived foods

#### 5.1.1 Meat and meat products

Meat (including pork, beef, and poultry) is susceptible to contamination by various pathogens during farming, slaughtering, and processing due to poor hygiene conditions. In addition, meat serves as an ideal substrate for microbial growth and reproduction because of its rich nutrients, neutral pH, and high water activity (Wu et al., 2018). Given this, many studies have investigated lytic enzymes as biocontrol agents to control the spread of pathogenic bacteria. Zhang F. et al. (2026) treated pork with 50 μg/mL of LytN at 37 °C for 6 h, achieving a 3.50 log_10_ CFU/mL reduction in methicillin-resistant *S. aureus* (MRSA). Similarly, LysPFX32 treatment of pork at 28 °C for 24 h effectively inactivated *Pseudomonas fluorescens* without altering the color or texture of the meat (Guan et al., 2025). In addition to their efficacy at room temperature, these enzymes are effective under low-temperature, and their activity is dose-dependent. Lu et al. (2023) demonstrated that under treatment conditions of 4 °C for 48 h, LysCP28 at 100 μg/mL and 50 μg/mL reduced *C. perfringens* in duck meat by 3.20 log_10_ CFU/g and 3.08 log_10_ CFU/g, respectively. Under the same conditions, 50 μg/mL of Lys572 reduced *Streptococcus suis* in pork by approximately 2 log_10_ CFU/mL while also effectively lowering the bacterial load during refrigerated thawing (Ning et al., 2026). Furthermore, some lytic enzymes (e.g., LysMR-5 and LysGH15) retain high antibacterial activity at high salt concentrations, which expands their range of applications in meat products (Kaur et al., 2020; Yan et al., 2021).

#### 5.1.2 Dairy products

As one of the most widely consumed dairy products, milk and its derivatives, such as cheese, butter, and milk powder, are highly susceptible to contamination by pathogenic bacteria, which pose a major threat to the dairy industry. Many studies have evaluated the application effects of endolysins and their combinations with other antimicrobial agents in dairy products. *Listeria*, as a common pathogen in milk, significantly hampers the development of the dairy industry. Holle and Miller (2021) demonstrated that PlyPSA, PlyP40, and PlyP100 reduced *Listeria* in fresh cheese during a 28-day refrigerated storage period. Furthermore, Zhang et al. (2025) demonstrated that LysP70 reduces the *Listeria* load in milk by up to 1.9 log₁₀ CFU/mL. Notably, the enzyme exhibited stronger lytic activity at 30 °C than at 4 °C, further expanding its application potential under different temperature conditions.

Lytic enzymes also exhibit significant antibacterial activity against other important milk borne pathogens, with this activity being influenced by milk components. Chang et al. (2017) first discovered that the combination of LysSA97 and carvacrol reduces methicillin-resistant *S. aureus* (MRSA) in milk, and its bactericidal effect in skim milk is significantly better than that in whole milk, which is attributed to the influence of fat on the binding of LysSA97 to MRSA cells. Ji Q. et al. (2026) and Ji Y. et al. (2026) further corroborated this conclusion, demonstrating that the endolysin alone also exhibited better bactericidal activity in skim milk than in whole milk. Similarly multiple lytic enzymes, such as PlyCP41, LysCPQ7 and LysCPD7, can inhibit the contamination and proliferation of *C. perfringens* in milk and cheese, demonstrating great potential as novel biocontrol agents against *C. perfringens* in dairy products and fermented foods (Swift et al., 2018; Mohammadi et al., 2024; Ha et al., 2026). Among them, LysCPQ7 significantly reduced the pathogen count in milk and sliced cheese at both refrigerated and room temperatures, with a greater effect in cheese (>4.5 log reduction) (Mohammadi et al., 2024). This is probably because the lysins, when evenly dispersed in a liquid matrix, experience a drop in effective concentration due to dilution and protein interactions.

#### 5.1.3 Eggs and egg products

Currently, the direct application of natural endolysins in egg products remains limited, yet their combination with permeabilizing agents enables the effective elimination of *Salmonella* contamination. For instance, Shen et al. (2023) treated eggshell surfaces with rLysJNwz plus EDTA for 60 min, achieving a bacterial reduction of over 86.7%. Similarly, the LysMD10 and citric acid (a food-grade organic acid) combination completely inactivated *Salmonella* on eggshells within 10 min, offering a safe and efficient biological decontamination strategy for egg processing (Ding et al., 2026). These findings demonstrate that permeabiliser-assisted endolysins can be used safely and efficiently to decontaminate eggs, and that the choice of permeabiliser significantly affects both efficacy and practical applicability.

#### 5.1.4 Seafood

*Vibrio parahaemolyticus* is a common and significant contaminant of seafood products. In the United States, *V. parahaemolyticus* causes an estimated 50,000 infections annually, with cases now regularly reported across Asia, Europe, and South America (Martinez-Urtaza and Baker-Austin, 2020). Faced with this global threat, early research efforts centered largely on phage development, while lysins have only recently emerged as a promising alternative. Some lysins, such as LysF23s1, LysVPB, and Lys59, have demonstrated potent *in vitro* activity against *V. parahaemolyticus* and other Gram-negative bacteria, showing great potential for applications in aquaculture and the control of seafood-related infections (Xia et al., 2022; Chen et al., 2025; Liu et al., 2025). Beyond these native lysins, engineered chimeric variants have also demonstrated considerable potential. Ning et al. (2021), for instance, fused a cationic peptide to the *V. parahaemolyticus* phage lysin Lysqdvp001 to generate a chimeric lysin (Lysqdvp001-15aa) that reduced the pathogen by 4.03 log_10_ CFU/g in oysters and effectively inhibited biofilm formation, thereby underscoring the practical value of engineered endolysins in seafood.

### 5.2 Lytic enzymes against pathogenic Bacteria in plant-derived foods

Fresh fruits and vegetables are important vehicles for foodborne pathogens, as they are often consumed raw or with minimal processing. Surface features such as stomata, cuts, and crevices provide bacteria with sheltered niches that allow them to evade conventional sanitizers. Various phage lysins have been evaluated for their ability to decontaminate vegetables. For example, Xu S. et al. (2021) assessed the capacity of PlyEc2 to eliminate Shiga toxin-producing *E. coli* (STEC) O157:H7 on romaine lettuce, reporting a 99.7% reduction of the pathogen. Similarly, the endolysin LysP70, derived from phage P70, significantly reduced the viable count of *L. monocytogenes* on lettuce (Zhang et al., 2025). However, most current studies have focused on short-term antibacterial effects at room temperature, while long-term efficacy remains suboptimal, likely due to the complexity of the food matrix and bacterial regrowth. Ionic strength has been shown to inhibit endolysin activity. For instance, treating lettuce with the endolysins cpp-Lys and Lys19 for 15 min significantly reduced *C. perfringens* counts, yet the addition of Zn^2+^ inhibited the activity of cpp-Lys (Zhao et al., 2023; Ji Y. et al., 2026). This further confirms that factors such as ionic strength can affect lytic enzyme activity, a matter that warrants attention during food storage and distribution.

Although phage lysins show considerable promise as antimicrobial agents in the food industry, research has largely focused on vegetables, with fruit receiving far less attention, likely because the inherently low pH of fruit tissues can suppress endolysin activity. However, not all endolysins are sensitive to low-pH environments. Notably, Lys-LP-XY2101 reduced *L. monocytogenes* to below the detection limit in apple juice (a low-pH fruit product) after 1 h of treatment at 4 °C, 16 °C, and 25 °C, with the best efficacy observed at room temperature (25 °C). This demonstrates that certain endolysins can remain highly active even in acidic fruit matrices (Ji Q. et al., 2026). Collectively, these findings highlight the great potential of endolysins for the biological control of pathogenic bacteria on fresh fruits and vegetables.

## 6 Application of lytic enzymes in agriculture

Plant pathogens are of significant economic importance, as they not only reduce crop yields but also threaten farmers’ livelihoods worldwide by causing severe losses in major crops. Common genera of bacterial pathogens in crops include *Xanthomonas*, *Pseudomonas*, *Erwinia*, and *Agrobacterium*. Expressing endolysins in plants via genetic transformation represents a potential strategy for suppressing bacterial growth. In an early study, transgenic potato plants expressing the T4 phage lytic enzyme were shown to acquire resistance to the plant pathogen *Erwinia carotovora* (Pirhonen et al., 1988). Subsequently, Wittmann et al. (2016) introduced the endolysin gene from phage CMP1 into tomato, obtaining transgenic lines resistant to the bacterial canker pathogen, which markedly reduced disease symptoms. Recently, Yao et al. (2025) reported LysPd078, the first endolysin shown to effectively control both Gram-negative and Gram-positive foodborne pathogens on mung bean seeds. This enzyme reduces the pathogen count by 4–5 log CFU, even when applied at low concentrations. Meanwhile, Nam and Kim (2026) fused the antimicrobial peptide Cecropin A (1–8) with the endolysin PlyPw to obtain Ceca^1-8^-PlyPw, which efficiently inhibits *Pectobacterium carotovorum*, the pathogen causing soft rot in Chinese cabbage. In postharvest leaf and seedling models, this fusion endolysin significantly alleviated soft rot symptoms and showed anti-*Pectobacterium* activity in soil, underscoring its potential as an alternative to agrochemicals throughout the crop supply chain and offering a new strategy for the biological control of plant diseases.

## 7 Application of natural lytic enzymes in biofilm removal

Bacterial biofilms are ubiquitous in the environment, forming highly structured communities when microorganisms attach to biotic or abiotic surfaces and secrete extracellular polymeric substances. These communities significantly diminish the effectiveness of disinfectants and antimicrobial agents, thereby posing a serious threat to food safety and public health. Of particular concern, biofilms formed by foodborne pathogens have become a critical issue in the food industry that urgently needs to be addressed. For example, *Clostridium perfringens* not only forms biofilms but also produces highly resistant spores, which confer significant tolerance to conventional disinfectants and oxidative stress (Hu et al., 2021). Unlike conventional agents, phage lysins possess a distinct cell wall hydrolysis mechanism, demonstrating great potential for eradicating such biofilms.

### 7.1 Effects on single-species biofilms

Many phage lysins effectively eradicate single-species biofilms on food contact surfaces, though their activity is both target-specific and material-dependent. For staphylococcal biofilms, LysCSA13 and LytN both show efficacy but differ markedly in their performance. LysCSA13 displays potent biofilm-removing activity across a variety of materials, including polystyrene, glass, and stainless steel (Cha et al., 2019), whereas LytN acts primarily on stainless steel, reducing viable MRSA 487 biofilm cells by 4.30 log_10_ CFU/mL within 6 h and outperforming its parent phage (Zhang F. et al., 2026). Moreover, their efficacy also varies significantly with the target *Staphylococcus* species. For instance, LysSA52 eliminates approximately 60% of *S. aureus* biofilms, while LysCP28 removes around 80% of *S. epidermidis* biofilms (Abdurahman et al., 2023; Lu et al., 2023). In contrast, LysSGF3 possesses a broader antibacterial spectrum and effectively reduces preformed biofilms of multiple Gram-positive and Gram-negative pathogens on microplates. Although its removal rate of approximately 50% is lower than those of the previous two enzymes against their specific targets, its broad-spectrum advantage remains evident (Chang et al., 2025). Similarly, LysBCC348 has been shown to reduce both the number of planktonic cells and the biofilm of *Bacillus cereus* on stainless steel and polystyrene surfaces (Seol et al., 2024). Furthermore, Lys19 and LysSS have been demonstrated to effectively inhibit or eradicate biofilms of *C. perfringens* and *Acinetobacter baumannii* (Ji Y. et al., 2026; Kim et al., 2020). Taken together, lytic enzymes demonstrate strong biofilm-inhibitory activity, underscoring their significant potential as surface disinfectants for food processing equipment.

### 7.2 Effects on multi-species biofilms

In both natural and food processing environments, biofilms are usually composed of multiple bacterial species. Owing to the synergistic interactions among community members, multispecies biofilms display enhanced tolerance to antimicrobial agents, making them more challenging to eradicate. Some endolysins exhibit broad-spectrum activity and can effectively disrupt multispecies biofilms. For instance, Ply113 eliminates dual-species biofilms composed of *S. aureus* and *Enterococcus faecalis* (Wang et al., 2023). Likewise, LysSYL specifically kills *S. aureus* while efficiently disrupting its dual-species biofilm with *A. baumannii*, achieving markedly greater efficacy than conventional antibiotics (Liu H. et al., 2024). Extending this potential to more complex communities, Lys22, an endolysin derived from a novel *Enterococcus* phage, effectively eradicates both single- and triple-species biofilms formed by *E. faecalis*, *S. aureus*, and *A. baumannii* (Yang et al., 2025). Although current research remains limited to laboratory-scale studies and has yet to be validated on food contact surfaces or within actual food matrices, these findings point to a promising new direction for food industry applications.

### 7.3 Enhancing anti-biofilm activity of lytic enzymes

To improve the anti-biofilm efficacy of lytic enzymes, synergistic strategies have garnered considerable interest. Hasan et al. (2024) demonstrated that in the presence of 50 mM lactic acid, LysEP114 and LysEP135 reduced biofilm cell counts from 7.3 log to 4.1 log and 2.2 log CFU/mL, respectively, suggesting that the endolysin-lactic acid combination holds promise for the simultaneous control of both planktonic and biofilm cells. Wang et al. (2025) combined the endolysin LysPEF1-1 with cinnamon bark essential oil (C.b.EO), which not only broadened the antibacterial spectrum but also demonstrated promising application potential on various food contact surfaces and vegetables. On polystyrene, stainless steel, and cabbage leaf surfaces, this combination treatment effectively removed the mixed-species biofilm formed by *L. monocytogenes* and *E. faecalis*, showing significantly better efficacy than either treatment alone, indicating that this combination has great potential for eliminating mixed-species biofilms on food and food contact surfaces.

## 8 Strategies for enhancing the efficacy of lytic enzymes

The modular nature of endolysins provides an ideal platform for their engineering. Currently, the main strategies for optimizing endolysins against Gram-positive bacteria include: (1) truncation or deletion of domains; (2) site-directed mutagenesis; and (3) domain swapping.

### 8.1 Strategies targeting lytic enzymes against gram-positive bacteria

#### 8.1.1 Truncation or deletion of domains

Domain truncation or deletion can modulate protein activity. Specifically, truncation of endolysins not only increases their lytic potency but also broadens their host range. For instance, Mayer et al. (2011) truncated the *Clostridium difficile* endolysin CD27L to its N-terminal domain (CD27L₁-₁₇₉), resulting in enhanced lytic activity and an expanded lytic spectrum that included strains previously insensitive to the full-length protein. Likewise, Choi et al. (2024) demonstrated that CHAPSAP26-161, a truncated variant retaining only the CHAP domain of LysSAP26, showed improved purification efficiency, stronger antibacterial activity, and a broad-spectrum antibacterial effect against *S. aureus*, *A. baumannii*, and *C. difficile*. In food applications, the amidase-deficient variant LysRODIΔAmi outperformed its parent endolysin LysRODI. While the latter reduced *S. aureus* counts in milk by only 1–2 log CFU, the former lowered bacterial levels below the detection limit within 15 min and was equally effective in fresh cheese, highlighting its potential for the biocontrol of *S. aureus* in milk (Agún et al., 2022). These findings suggest that smaller proteins typically exhibit faster diffusion and more efficient target penetration. Consequently, rational domain truncation represents a viable strategy for optimizing endolysin performance. However, the functional outcomes of domain removal or truncation remain largely unpredictable. Although most studies have demonstrated that domain truncation enhances endolysin activity, some have reported that domain removal may compromise protein function or selectivity (Yu et al., 2021; Krishnan et al., 2025).

#### 8.1.2 Site-directed mutagenesis

Substituting the active site or key amino acid residues can enhance both the catalytic efficiency and thermal stability of endolysins (Khan et al., 2023; Zhang Y. et al., 2026). This rational design strategy, grounded in protein engineering, represents an effective route for improving endolysin properties. Low et al. (2011) suggested that increasing the net charge of the catalytic domain of endolysins through mutating surface residues to positively charged amino acids represents a direct strategy for fine-tuning or expanding the lytic host range. However, Shang and Nelson (2019), later noted that simply increasing the net charge is insufficient to confer cell wall-binding domain (CBD)-independent lytic activity. Thus, while the charge hypothesis should be an important consideration in future engineering efforts, it should not be the sole focus. In addition to charge modification, other site-directed mutagenesis strategies have yielded favorable outcomes. For instance, a recent study showed that mutating a non-catalytic gating residue (H37A) within the catalytic cavity of T7 amidase enhanced enzymatic activity by approximately 35% and optimized the balance between stability and activity (Tyagi et al., 2023). Although this study focused on Gram-positive bacteria, the same mutagenesis strategy is also applicable to Gram-negative bacteria. For instance, mutating a single residue within the hydrophobic core enhanced the thermostability of LysF1 without affecting its enzymatic activity (Love et al., 2021).

#### 8.1.3 Domain swapping

The modular structure of endolysins enables the construction of chimeric endolysins with altered properties through domain shuffling or replacement. Compared with mutagenesis and active-site modification, this domain-level engineering strategy provides greater flexibility and design freedom. To date, chimeric endolysins have been successfully applied in milk and raw meat for pathogen control. Mao et al. (2013) constructed a chimeric protein by fusing the endopeptidase domain of Ply187 to the SH3b cell wall-binding domain of LysK. This chimeric endolysin demonstrated markedly improved lytic activity in milk. Beyond single fusions, the construction of hybrid libraries represents an efficient and rapid strategy. Lee et al. (2021) generated a hybrid endolysin library by exchanging the enzymatic activity domains (EADs) and cell wall-binding domains (CBDs) of 12 natural staphylococcal endolysins, from which a novel chimeric endolysin, ClyC, was screened. ClyC demonstrates strong antibacterial activity against *S. aureus* in milk and can effectively eradicate biofilms formed by multidrug-resistant strains. In another study, by shuffling the domains of LysCPD9 with those of the thermostable *C perfringens* endolysin LysCPS2, a novel chimeric endolysin, ClyY, was constructed. With greater antibacterial activity than its parent, reducing *C. perfringens* counts in artificially contaminated milk and beef by 5-log CFU/mL and 3-log CFU/cm^2^, respectively. This finding extended the application of chimeric endolysins to beef, in addition to milk (Choi et al., 2023).

### 8.2 Strategies targeting lytic enzymes against gram-negative bacteria

The outer membrane of Gram-negative bacteria acts as a formidable barrier that prevents macromolecular lytic enzymes from reaching the peptidoglycan layer, thereby impeding bacterial lysis. This limitation greatly restricts the use of lytic enzymes in food decontamination. Consequently, multiple strategies have been devised to circumvent this barrier.

#### 8.2.1 Combined application strategies with outer membrane permeabilizers

The outer membrane consists mainly of lipopolysaccharides and phospholipids. To date, multiple strategies have been developed to circumvent this barrier, which, based on their mechanisms of action, can be categorized into chelating agents, organic acid outer membrane disruptors, and polycationic compounds (see Table 2). Chelating agents destabilize the lipopolysaccharide layer by binding divalent metal ions, thereby disrupting the outer membrane and enabling lysins to reach the peptidoglycan layer. Taking EDTA as an example, its combination with endolysins LysP6 or LysSP1 has been shown to markedly enhance the bactericidal activity against *Salmonella* (Jiang et al., 2021; Wang et al., 2025). Nevertheless, due to its strong anticoagulant properties, EDTA raises potential safety concerns in food products, restricting its practical application. Furthermore, weak organic acids commonly used as food additives, such as lactic acid, citric acid, and malic acid, utilize their acidity to acidify the local environment, thereby increasing outer membrane permeability and facilitating the lytic activity of endolysins against Gram-negative bacteria. For instance, Sisson et al. (2024) demonstrated that combining PlyPa03 with citric acid offers an effective strategy for controlling *Pseudomonas syringae* infections in kiwifruit orchards. Li et al. (2025b) showed that LysBT1, assisted by either EDTA or citric acid, could kill *E. coli*; however, only citric acid enabled the killing of *A. baumannii*, while neither agent was effective against *Pseudomonas aeruginosa*. Collectively, these results suggest that the bactericidal efficacy of endolysins depends on the strain and the type of permeabiliser used.

Polycationic compounds, such as polymyxins, poly-L-lysine, and aminoglycosides, function through electrostatic interactions, either by competitively displacing divalent cations from lipopolysaccharides or by directly inserting into the outer membrane and disrupting its architecture, thereby acting synergistically with endolysins to enhance antibacterial efficacy (Ning et al., 2021; Kim J. et al., 2022; Soontarach et al., 2025). Beyond the aforementioned approaches, the combination of endolysins with membrane-active natural products, such as essential oils, which are secondary metabolites of plants, has demonstrated synergistic antibacterial activity, thereby paving the way for the design of innovative food preservatives and the enhancement of food safety and quality (Chang et al., 2017; Kim et al., 2024; Wang et al., 2025).

#### 8.2.2 Engineered endolysins

The fusion of endolysins with outer membrane-penetrating peptides has led to the successful development of engineered variants, including artilysins, innolysins, and lysocins, which exhibit markedly enhanced antibacterial activity against Gram-negative bacteria (Kocot et al., 2023). Artilysins are primarily designed by fusing an outer membrane-penetrating peptide, which allows the chimeric protein to cross the outer membrane, access the peptidoglycan layer, and degrade it, resulting in markedly improved bactericidal activity. Their mechanism involves outer membrane disruption or penetration, self-promoted uptake into the periplasmic space, and subsequent peptidoglycan cleavage (Kim et al., 2025). This strategy not only overcomes the inherent inability of native endolysins to act against Gram-negative bacteria but also offers a novel route to combat multidrug-resistant Gram-negative pathogens, holding considerable promise for both local infection therapy and food-borne pathogen control (Briers et al., 2014; Xu D. et al., 2021). Mortazavi et al. (2025) demonstrated that the artilysin PVP-SE1 effectively eradicates 48-h biofilms formed by various bacterial species on polystyrene surfaces. Moreover, it significantly reduces the counts of *Salmonella Typhimurium* and *L. monocytogenes* in low-fat UHT milk at 25 °C, highlighting its considerable promise as a biocontrol agent for dairy processing environments. However, existing research has predominantly depended on laborious and time-intensive individual screening approaches. To overcome this limitation, alternative strategies have been explored. Zeng et al. (2024) constructed a recombinant artilysin library containing 38 antimicrobial peptides and 8 endolysins. By employing rapid activity-based screening, they successfully pinpointed three highly efficient artilysins. This strategy not only substantially enhanced the efficiency of discovery but also generated molecules with exceptionally potent bactericidal activity against both *P. aeruginosa* and its biofilms.

Furthermore, innolysins exert their antibacterial effects by coupling enzymatic activity with the targeting ability of phage receptor-binding proteins (RBPs), enabling precise delivery to Gram-negative bacteria and targeted disruption of membrane architecture. For instance, innolysin Cj1 and Cj5, generated by fusing the H-fiber of a *Campylobacter jejuni* prophage with T5 endolysin, reduced *C. jejuni* loads on chicken skin by 1.63 log and 1.18 log, respectively, at 5 °C, highlighting a promising biocontrol approach for ensuring food safety during cold-chain storage (Zampara et al., 2021). Furthermore, innolysins hold promise for controlling bacterial contamination in fermented and dairy products. By targeting a broad spectrum of pathogenic bacteria, they can improve food safety while preserving overall product quality (Kim et al., 2025).

Like innolysins, lysocins (such as colicin) are constructed by fusing an endolysin with the translocation domain of a bacteriocin, leveraging the bacteriocin’s inherent outer-membrane translocation machinery to achieve targeted delivery. Consequently, lysocins display both high specificity and strong antimicrobial efficacy. Heselpoth et al. (2019) generated the lysocin PyS2-GN4 by fusing the receptor-binding and translocation domains (I–III) of the S-type pyocin PyS2 with the GN4 phage lysin. This construct actively delivers the lysin into the periplasm of *P. aeruginosa*, resulting in potent bactericidal activity (MIC as low as 0.25 μg/mL) and effective biofilm disruption.

### 8.3 Encapsulation enhances the stability of lytic enzymes

Liposomal encapsulation has been shown to significantly enhance drug stability and targeted delivery for antitumor applications (Li et al., 2017; Wang et al., 2021; Zhang et al., 2021; Chang et al., 2023; Jia et al., 2023). Inspired by these successes, various encapsulation techniques have recently been adopted in the food industry for bacteriophage lysins, which protects them from detrimental factors in food matrices and thereby extends their antimicrobial activity. Among these techniques, liposomes represent one of the most extensively applied formulation platforms. Encapsulation within liposomes safeguards endolysins against chemical degradation, thereby preserving their stability and activity throughout transportation and storage (Zhang Y. et al., 2026). For instance, encapsulation of the endolysin BSP16Lys in cationic liposomes reduced the titers of *Salmonella Typhimurium* and *E. coli* by 2.2 log and 1.6 log CFU/mL, respectively, without the need for a membrane permeabilizer (Bai et al., 2019). This encapsulation strategy was also effective against Gram-positive bacteria. Portilla et al. (2020) incorporated LysRODI into pH-sensitive liposomes, achieving a reduction of >2 log CFU/mL against both planktonic cells and biofilms of *S. aureus* (including MRSA) when tested at pH 5. This work validated the feasibility of liposome-mediated targeted delivery in acidic microenvironments. In another study, Ghate and Poluri (2025) encapsulated the endolysins T4L and T7L in calcium alginate beads, which enabled pH-dependent release and thereby offered a practical approach for targeted endolysin delivery in acidic food matrices. Beyond the aforementioned encapsulation strategies, other carriers such as nanoparticles (e.g., chitosan) and even probiotics have been reported to improve the lytic activity of endolysins against Gram-negative bacteria (García et al., 2010; Chun et al., 2020; Vázquez et al., 2021; Ramírez Saenz et al., 2024). Chitosan nanoparticles represent highly promising, biocompatible delivery platforms capable of improving the bioavailability and extending the half-life of endolysins (Vázquez et al., 2021). For instance, incorporating the endolysin LysST-3 into chitosan nanoparticles (CS-NPs) to generate LysST-3-CS-NPs led to marked enhancements in stability, reusability, and anti-biofilm capacity, thereby offering a new strategy for controlling bacterial infections in the food and healthcare sectors (Liu B. et al., 2024). Probiotics represent a distinctive class of live delivery vehicles with considerable application potential. In contrast to the passive protection afforded by liposomes and nanoparticles, probiotics can be genetically engineered for the *in situ* production, active secretion, or surface display of endolysins, thereby enabling a sustained antibacterial action within complex environments such as the gastrointestinal tract (Gervasi et al., 2014; Chun et al., 2020; Moreno et al., 2026). This “living factory” concept offers a novel strategy for the deployment of endolysins in food applications.

Encapsulation is particularly relevant for food applications because it can reduce enzyme exposure to unfavorable pH, salts, proteases, fats, and other matrix components, while also improving local delivery to bacterial cells on food surfaces or within biofilms (Maleki et al., 2022; Zabot et al., 2022; Paidari et al., 2024). Accordingly, delivery systems should be evaluated not only for immediate bactericidal activity but also for enzyme retention, release kinetics, storage stability, and compatibility with sensory quality.

### 8.4 Computer-aided and bioinformatics mining

Conventional molecular screening approaches are not only cumbersome and time-consuming but also tend to yield native endolysins with inherently limited lytic activity. By contrast, the integration of artificial intelligence and bioinformatics enables high-throughput screening and rapid discovery of potent endolysin candidates, thereby substantially enhancing screening efficiency and the quality of research and development.

A proper assessment of the antibacterial potential of endolysins necessitates the systematic characterization of their biochemical and biological properties. In recent years, a number of machine learning-driven bioinformatics tools, including Lypred, CWLy-SVM, CWLy-pred, and CWLy-RF, have been established to differentiate lytic from non-lytic proteins based on amino acid frequency and sequence order (Chen et al., 2016; Meng et al., 2020a, 2020b; Jiao et al., 2021). Nevertheless, these tools largely depend on characteristics derived from known lytic proteins, and their predictive accuracy for structurally novel or evolutionarily distant lytic proteins still requires enhancement. To overcome this limitation, Criel et al. (2021) carried out a comprehensive survey and established the PhaLP database, which compiles over 16,000 phage lytic protein sequences. By integrating this database with machine learning, they uncovered domain-level design principles that determine endolysin host specificity, thus offering a robust bioinformatics platform for the high-throughput discovery of highly active endolysins. Building on that, Nandy et al. (2022) employed bioinformatics tools and computational approaches to identify 14 antimicrobial peptides from eight endolysins of *A. baumannii* phages. Several of these peptides exhibited antibiofilm, antifungal, and cell-penetrating activities, and none showed cytotoxicity, demonstrating an efficient computational strategy for the discovery of novel antimicrobial agents. Future consortia could create standardized, custom databases with consistent ontologies and naming, integrate scattered sequence information, and include biochemical, molecular, and evolutionary data (e.g., domains, families) to further boost screening efficiency (Bałdysz et al., 2024).

## 9 Current challenges in the application of lytic enzymes

### 9.1 Practical challenges in screening high-activity lytic enzymes

The discovery of highly active endolysins from bacteriophages involves multiple steps: phage isolation and purification, genome sequencing and annotation, followed by cloning, expression, and functional validation of candidate endolysins. Although the workflow is clear, several practical challenges persist: genome annotation relies heavily on homology mapping, some endolysins exhibit poor solubility or low activity, and screening for endolysins from existing sequence databases requires extensive experimental validation. Moreover, even when endolysins are designed *de novo* using artificial intelligence, their antibacterial activity must still be confirmed through antimicrobial assays (Zheng and Zhang, 2024).

### 9.2 Insufficient activity and stability in food matrices

Despite substantial evidence demonstrating the potent bactericidal activity of endolysins across various food matrices, most studies remain confined to small-scale laboratory settings, and activity observed in buffer systems may not translate directly to complex foods. As proteinaceous antimicrobials, endolysins are highly susceptible to processing and storage variables, including pH, temperature, ionic strength, water activity, proteolytic enzymes, and food matrix composition (Schmelcher and Loessner, 2016; Shannon et al., 2020; Kim et al., 2025). Acidic environments, low temperatures, high salt concentrations, and repeated temperature fluctuations can cause protein denaturation or reduce catalytic efficiency. Food components such as fats, proteins, salts, and divalent ions may interfere with enzyme diffusion, substrate recognition, binding to the bacterial cell wall, or catalytic activity (Chang et al., 2017; Shannon et al., 2020; Cui et al., 2025). Previous studies have shown that the endolysins LysAB54 and PlyA lose all antibacterial activity in complex media such as LB, serum, and milk, likely because ionic interactions or matrix components neutralize their activity (Yang et al., 2015; Khan et al., 2021). Physical properties of food matrices can also compromise efficacy (Schmelcher and Loessner, 2016; Shannon et al., 2020). For example, irregular surfaces, stomata, crevices, biofilm extracellular polymeric substances, and viscous liquid products may limit direct contact between the enzyme and target bacteria. Taj et al. (2025) observed that the antibacterial performance of lysSEP21 varied with food surface type: while it showed good bactericidal effects on beef, chicken, and lettuce, its efficacy in liquid whole egg was considerably lower, likely due to the viscous texture of egg liquid hindering direct enzyme-pathogen contact. Therefore, future studies should assess lysin stability and antimicrobial activity under realistic processing and storage conditions, including refrigerated storage, acidic or high-salt products, heterogeneous food surfaces, mixed microbial communities, and repeated contamination or regrowth scenarios.

### 9.3 High production cost

As enzymatically active proteins, lytic enzymes require standardized fermentation, purification, and activity testing procedures for large-scale application (Zheng and Zhang, 2024). Consequently, their commercial adoption in the food industry faces a high-cost barrier, especially within complex food systems where further investigation is needed to enhance and optimize their stability and activity.

### 9.4 Consumer acceptance

Consumer acceptance is a key determinant of the real-world applicability of these substances in food production. The majority of consumers prefer natural, minimally processed foods and have reservations about endolysins derived from viruses. Application methods such as spraying or coating may be perceived negatively, and trust in their antimicrobial efficacy and safety remains limited. Therefore, efforts should be made to enhance public understanding of the origins and mechanisms of endolysins, conduct rigorous safety evaluations, and devise consumer-oriented food-grade delivery systems, such as edible films or natural carrier encapsulation, to progressively foster public acceptance.

### 9.5 Standardization and regulatory considerations

A further barrier to practical deployment is the lack of standardized reporting frameworks for lysin efficacy in foods (Wang et al., 2026). For regulatory evaluation, application studies should report the initial inoculum, lysin concentration or dose, application frequency, treatment time, treatment temperature, food matrix, application mode, storage duration, and the primary antimicrobial endpoint. The preferred endpoint for planktonic cells should be log_10_ CFU reduction relative to untreated controls, supported by statistical analysis across biological replicates. For biofilm studies, viable biofilm-cell reduction should be reported separately from total biomass reduction, because these measurements describe different biological outcomes. When percentage reduction, time to detection limit, spoilage delay, or sensory quality is reported, these outcomes should be clearly distinguished from culture-based log reductions.

Regulatory authorities are likely to require a reproducible data package that includes enzyme identity and purity, production host safety, absence of toxic contaminants, absence of virulence or antimicrobial-resistance genes in the production system, toxicological and allergenicity assessment, dose–response relationships, target-pathogen spectrum, matrix-specific efficacy, stability during processing and storage, residue or degradation profile, effects on sensory quality, and compatibility with existing food-processing workflows (Lu et al., 2023; Cui et al., 2025; Wang et al., 2026). Such standardized datasets would strengthen comparisons across studies and support the transition of lysins from laboratory-scale biocontrol candidates to validated interventions for foodborne pathogen control.

## 10 Conclusions and future prospects

Phage lysins, as phage-encoded peptidoglycan hydrolases, have emerged as a research focus in the field of food biocontrol due to their rapid action, high specificity, safety, and low resistance risk. This review systematically summarizes the progress in applying lysins to control foodborne pathogens, covering their structural characteristics, mechanisms of action, application efficacy in various food matrices, biofilm removal capabilities, and the multiple engineering and delivery strategies developed to enhance their antibacterial activity. Although numerous studies have confirmed that lysins exhibit significant inhibitory effects against various pathogens under laboratory conditions and show promising application potential in real food systems such as meat, dairy products, and vegetables, their widespread adoption in the food industry still faces multiple challenges. These include difficulties in screening highly active lysins, reduced activity and stability in food matrices, high production costs for large-scale manufacturing, the need for standardized efficacy endpoints and regulatory data packages, and consumer concerns regarding the viral origin of these enzymes. Future research should focus on the following areas: (1) Leveraging bioinformatics and artificial intelligence technologies to establish standardized databases and high-throughput screening platforms, thereby accelerating the discovery of novel lysins. (2) Combining protein engineering with encapsulation technologies to improve the stability and targeting of lysins in complex food environments. (3) Conducting systematic food safety, toxicological, allergenicity, and matrix-specific efficacy evaluations using harmonized endpoints such as log10 CFU reduction, biofilm viable-cell reduction, pathogen regrowth during storage, and sensory quality. (4) Developing corresponding regulatory standards that specify enzyme identity, purity, dose–response relationships, production-system safety, stability, and degradation or residue profiles, and (5) Strengthening public education and transparent communication to increase consumer acceptance of lysins as natural, safe, and efficient biopreservatives. In summary, lysins hold great promise as next-generation biopreservatives for food. Their continued research and application will effectively drive the strategic transformation of food safety from chemical-based control to green biocontrol approaches.
